# Quantitative assessment of systolic and diastolic left ventricular twist using Fourier Analysis of STimulated echoes (FAST) and CSPAMM

**DOI:** 10.1186/1532-429X-14-S1-P240

**Published:** 2012-02-01

**Authors:** Meral Reyhan, Daniel Ennis

**Affiliations:** 1 Radiological Sciences, Univerisity of California, Los Angeles, CA, USA; 2 Biomedical Physics Interdepartmental Program, Univerisity of California, Los Angeles, CA, USA

## Summary

The FAST method for measuring left ventricular twist has been expanded to semi-automatically measure torsion, peak systolic twist rate, peak diastolic untwisting rate, time to peak twist, and duration of untwisting using FAST+CSPAMM.

## Background

Alterations in left ventricular (LV) twist are important for many pathologies including aortic stenosis[1], diastolic dysfunction[2] and aging[3]. LV twist is a measure of the rotation of the apex relative to the base of the heart. Employing a previously validated method, Fourier Analysis of STimulated echoes (FAST)[4], which measures object rotation directly in Fourier space, we performed quantitative analysis of LV twist, torsion, twisting rates, time to peak twist, and duration of untwisting in thirteen (n=13) healthy volunteers using SPAMM and CSPAMM[5] tagged images. FAST analysis typically requires 2-3 minutes of user interaction time.

## Methods

A spoiled gradient echo pulse sequence was modified to support CSPAMM and used to acquire short-axis images in healthy volunteers (n=13) at the LV base and apex with the following parameters: 360-300x300-280mm FOV, 5-6mm slice thickness, 192x144 acquisition matrix, 501 Hz/pixel receiver bandwidth, TE/TR=3.5-3.7/4.7-6.5ms, 10mm tag spacing, 7-8 ky-lines per segment, 3/4 partial Fourier imaging, 14-16 cardiac phases, 15° FA and two averages for SPAMM imaging, and non-linearly ramped imaging flip angles (initial angle 22°) for CSPAMM[6]. Estimates of LV rotation at basal and apical slice levels for all frames were obtained from the rotation of the stimulated echo and stimulated anti-echo about the FID in Fourier Space using the FAST method[4]. Bland-Altman analysis, linear regression, paired t-test, and Wilcoxon signed-rank test were used to compare FAST+CSPAMM to a previously validated FAST+SPAMM technique. Only data obtained during the first 500ms was used for comparison of SPAMM and CSPAMM due to tag fading. P<0.01 was considered statistically significant.

## Results

The comparison of FAST+CSPAMM to FAST+SPAMM for twist, torsion, twisting rates, time of peak twist and duration of untwisting are summarized in Table 1. Table 2 provides peak rotation and time to peak rotation for systole and diastole for the apical and basal slices, which are important metrics in aortic stenosis[1] and aging[3]. No significant differences were found for twist, torsion, or twist rate measurements made with FAST+CSPAMM to FAST+SPAMM using the paired t-test or the Wilcoxon signed-rank test. Bland-Altman analysis and linear regression demonstrate excellent agreement between techniques (Table 1). These values match well with literature[7].

**Table 1:** Comparison of FAST+CSPAMM versus FAST+SPAMM for mean peak twist, torsion, systolic twisting rate, diastolic untwisting rate, normalized untwist rate, time to peak systolic twist, and duration of untwist.

	FAST+CSPAMM	FAST+SPAMM	Bland-Altman	Linear Regression
	Mean Peak Value	Mean Peak Value	Bias, [95%CI]	CSPAMM=m*SPAMM, r

Twist [deg]	12.7±3.0	12.0±3.3	0.2, [-5.6,6.0]	CS= 0.98*SP, r=0.8
Torsion [deg/cm]	3.2±0.9	3.0±0.8	0.08, [-1.3,1.4]	CS= 0.99*SP, r=0.83
Systolic Twisting Rate [deg/s]	81.8±38.2	69.9±20.7	-0.6, [-67.3,66.2]	CS= 0.90*SP, r=0.84
Diastolic Untwisting Rate [deg/s]	-102.7±24.6	-106.7±32.4		
Normalized Untwist Rate [1/s]	-8.4±2.4	-9.3±2.6		
Time to Peak Systolic Twist [ms]	278.5±22.9	293.0±24.9		
Duration of Untwisting [ms]	147.9±21.0			

Peak twist was defined as the maximum twist value for each subject. LV torsion was calculated as LV twist divided by the distance between the apical and basal slices with units of deg/cm. Peak torsion was defined as the maximum torsion value for each subject. Twist rate was calculated as the discrete derivative of twist with respect to time with units of deg/s. Peak twist rate was defined as the maximum twist rate. Peak diastolic untwist rate was defined as the minimum twist rate. Normalized peak untwist rate was defined as the minimum twist rate divided by the maximum twist angle with units of s-1. Time to peak twist was defined as the time [ms] from the first image frame (t=0 ms) until the frame of peak twist. To determine the duration of untwisting the data was normalized and interpolated. Cubic smoothing spline interpolation to 1% increments of the cardiac cycle was performed. This step was done to reduce noise and better detect the end of untwisting, which was determined by finding the maximum of the second derivative of the twist data after peak systolic twist.

**Table 2:** Summary of peak rotational values, peak diastolic rotation rate, and their corresponding times for the apical and basal slices.

	FAST+CSPAMM	FAST+SPAMM
Peak systolic rotation (base) [deg]	-4.8±2.5	-4.5±3.0
Time of peak [ms]	357.6±34.5	393.4±96.6
Peak diastolic rotation rate (base) [deg]	62.5±34.3	55.5±39.9
Time of peak [ms]	470.3 ±68.7	474.6 ±84.8
Peak systolic rotation (apex) [deg]	8.7±2.9	7.9±2.6
Time of peak [ms]	291.9±59.3	301.7±78.3
Peak diastolic rotation rate (apex) [deg/s]	-74.2±29.5	-69.0±34.4
Time of peak [ms]	417.1±33.7	417.7±76.6

## Conclusions

The FAST+CSPAMM is a semi-automated method that provides a quick and quantitative assessment of systolic and diastolic left ventricular twist, torsion, and twisting rates. When FAST is used in conjunction with CSPAMM duration of untwisting can be measured, which may provide further insight into left ventricular diastolic dysfunction.

## Funding

AHA to MLR.
